# Optimizing and tailoring cold atmospheric plasma parameters for *C. albicans* biofilms eradication

**DOI:** 10.3389/fmicb.2026.1786008

**Published:** 2026-04-10

**Authors:** Manca Lunder, Sebastian Dahle, Rok Fink

**Affiliations:** 1 Faculty of Health Sciences, University of Ljubljana, Ljubljana, Slovenia; 2 Faculty of Biotechnical, University of Ljubljana, Ljubljana, Slovenia

**Keywords:** applied voltage, *Candida albicans*, cold atmospheric plasma, fungicidal, treatment duration

## Abstract

**Aim:**

This study aims to evaluate the effect of applied voltage and treatment duration of cold atmospheric plasma (CAP) against *Candida albicans* biofilms.

**Methods:**

We evaluated the effects of CAP input voltage and treatment duration on inhibition zones, biofilm viability and metabolism, cell membrane integrity, biofilm oxidative stress, biomass composition, and hyphal structure.

**Results:**

Higher applied voltages and longer treatment duration significantly reduced biofilm viability, with complete eradication was achieved after 3 min at 16 and 20 V, while longer exposure (5 min) was required at lower voltages (8 and 12 V). The results show a clear response relationship between CAP treatment parameters and biofilm eradication. The assessment of cell membrane integrity supports the results, as membrane damage increases with increasing treatment parameters. Biofilm oxidative stress increased with treatment duration and morphological examination shows contraction and compression of the hyphal structure. Molecular changes in biofilm biomass show significant changes in lipids, proteins and carbohydrates depending on CAP settings.

**Conclusion:**

This study emphasizes the potential of optimal CAP treatment parameters as a promising approach to control *C. albicans* biofilms and highlights the complex dynamics of their effects on biofilm viability, morphology and composition.

## Graphical Abstract

**Figure:**
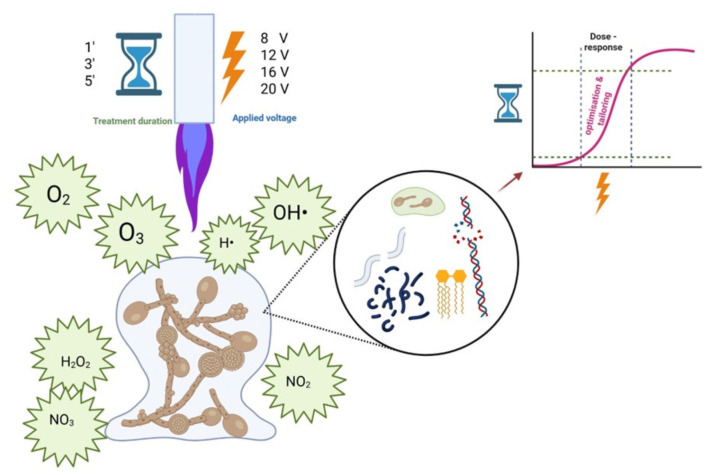
The evaluation of cold atmospheric plasma for *C. albicans* biofilm eradication (created with BioRender.com).

## 1 Introduction

Fungal infections, particularly from resistant *Candida* strains, pose a significant public health concern (Parambath et al., 2024). Globally, *Candida* spp. represent a major source of healthcare-associated infections, and *Candida* bloodstream infections carry an estimated 30% risk of mortality (Aslanov et al., 2025). *C. albicans*, a major cause of oral and genital infections is part of the normal flora in mucus membranes (Rahimi-Verki et al., 2016), however, it can cause life-threatening systemic infections in immunocompromised patients, with mortality rates reaching up to 50% (Chiodi Borges et al., 2017). *C. albicans*, one of the most predominant clinically isolated species, has virulence factors like dimorphism, which allows it to form biofilms on the surface with various morphological forms, e.g., yeasts, pseudo hyphae, and hyphae (Lee et al., 2024). Fungal biofilms are significantly more resistant to antifungals, being up to 20 times less susceptible to amphotericin B and 100 times less to fluconazole in comparison to planktonic cells (Maisch et al., 2012). For example, *C. albicans* can adhere to various surfaces, which facilitates its persistence in hospital environments and contributes to the formation of deleterious biofilms (Cavalheiro and Teixeira, 2018; Handorf et al., 2018). Although antifungals like fluconazole target *C. albicans* by disrupting ergosterol biosynthesis and compromising the cell wall, ultimately leading to cell death (Atriwal et al., 2021), their effectiveness is decreasing due to adverse side effects and fungal resistance (Rahimi-Verki et al., 2016). Therefore, science is urged to find new antibiofilm agents that are efficient, safe and do not cause resistance. One of the potentials is Cold Atmospheric Plasma (CAP), as innovative, non-toxic and cost-effective approach (Mirza Alizadeh et al., 2018). CAP is produced by applying high voltage to gas, creating charged particles, reactive species, and photons (Mai-Prochnow et al., 2014; Shapourzadeh et al., 2016; Modic et al., 2017) that have an impact on different microorganisms, e.g., bacteria, fungi, and viruses (Polito and Kushner, 2024). Its antimicrobial properties stem from reactive oxygen and nitrogen compounds (RONS), including superoxide anion radical (O_2_^–^), hydroxyl radical (OH), hydrogen peroxide (H_2_O_2_), nitric oxide (NO), nitrate (NO_3_^–^) and peroxynitrite anion (ONOO^–^) (Flynn and Gilmore, 2018), as they can etch cell membranes and interact with intracellular molecules, such as DNA and proteins (Chiodi Borges et al., 2017). For example, Morais et al. (2025) applied CAP on sterilized titanium alloy for dental implants and showed prevention of *C. albicans* pseudohyphae formation. Another study showed that CAP can disrupt the membrane redox systems of the *Trichophyton rubrum* fungus, leading to potassium ion efflux, cell lysis, and cellular death (Shapourzadeh et al., 2016). It also reduces yeast spread in infected skin models, inhibits hyphal growth, and decreases inflammation (Fink et al., 2023). Unlike antifungal agents that require specific cell interactions, CAP induces general oxidative and radical damage (Maisch et al., 2012). Chiodi Borges et al. (2017) tested helium-based CAP on *C. albicans* and found a decrease in virulence factors and lower adherence to human fibroblasts. CAP’s fungicidal effect was also demonstrated in a study by Kostov et al. (2015) that tested the planktonic form of clinical strains of *C. albicans* against He/O_2_ based CAP. Furthermore, Nishime et al. (2017) examined different bacterial and fungal strains and found that *C. albicans* was the least susceptible to helium-based CAP treatment. However, the interactions of CAP with *C. albicans* and its effectiveness in biofilm eradication remain poorly understood. Although some studies have addressed yeast disinfection with CAP, they offer limited insight into its effects on *C. albicans* biofilms and the mechanisms by which CAP treatment parameters influence biofilm removal. Therefore, the aim of this study was to assess how the effects of CAP applied voltage and treatment duration facilitate *C. albicans* biofilm eradication. Specifically, we investigated: (i) CAP inhibition screening on planktonic cells (ii) biofilm viability and metabolism (iii) cell membrane integrity and intracellular stress (iv) biomass assessment. Our goal is to optimize CAP parameters to improve the response efficiency of biofilm eradication to CAP parameters, which will advance disinfection methods *C. albicans*.

## 2 Materials and methods

### 2.1 Model microorganisms

The reference strain of *C. albicans* ATCC 10231 was obtained from the cryogenic preservation collection of the Faculty of Health Sciences of the University of Ljubljana. The strains were cultivated on Sabouraud maltose agar and incubated at 37°C for 48 h prior to analysis.

### 2.2 Plasma jet device

Plasma treatments utilized a gliding arc CAP jet device, detailed in prior studies (Kariž et al., 2021; Dahle et al., 2022) and shown in Supplementary Appendix 1. It has been adapted for the treatment of biofilm in microtiter plates, with the CNC programing. The device was equipped with an 8 mm modified aluminium nozzle, coated with epoxy resin with two 1.5 mm diameter copper electrodes. The electrodes were spaced 2 mm apart; each connected to a high-voltage power source via a wire (Dahle et al., 2022). Atmospheric air was used, as it is environmentally acceptable, compared to the use of noble gases, and is also less expensive. The presence of both ROS and RNS enhances the antimicrobial action (Katsigiannis et al., 2022). Air at a 35 L/min flow rate was supplied via an internal pump through a polypropylene tube (Dahle et al., 2022). The device operated at input voltages of 8, 12, 16, and 20 V, with power consumption of 20.4, 30.5, 41.7, and 56.8 W, respectively (Dahle et al., 2022). The surface temperature of the CAP was below 30°C at all times (Supplementary Appendix 2).

### 2.3 *C. albicans* inhibition zones screening

The initial potential of CAP was tested for its inhibition potential on solid media. Overnight cultures of *C. albicans* on Sabouraud maltose agar were suspended in a 0.9% NaCl solution and adjusted to 0.5 McFarland. Then 100 μL of the suspension was spread on Sabouraud maltose agar and allowed to dry for 20 min at room temperature. In the next step, the plates were treated with CAP at a distance of 1 mm from the bottom of the CAP jet (shown in Supplementary Appendix 1) and a treatment time of 1, 3, or 5 min at 8, 12, 16, or 20 V, respectively. The plates were then incubated at 37°C for 24 h. Inhibition zones were measured in four replicates and two trials.

### 2.4 Cold plasma anti-biofilm assay

Overnight cultures of *C. albicans* on Sabouraud maltose agar were suspended in a 0.9% NaCl solution (Merck KGaA, Germany), adjusted to 0.5 McFarland (1.5 × 10^8^ CFU mL^–1^) and diluted to 5 × 10^5^ CFU mL^–1^ in nutrient broth (Biolife, Italy). A total of 300 μL aliquot was transferred to 24-well microtiter plates (Nunc, Denmark) and incubated in static conditions at 37°C for 48 h. The culture medium was refreshed after first 24 h of incubation. After washing three times with 300 μL phosphate-buffered saline (PBS), the biofilms were allowed to air-dry at room temperature for 20 min. This drying period was carefully controlled to avoid excessive desiccation and did not result in a detectable reduction in viable cell numbers. The biofilms were then treated separately with CAP for 1, 3, or 5 min at 8, 12, 16, or 20 V input voltage and 1 mm spacing from the bottom of the CAP jet to the biofilm surface. Untreated biofilms served as controls, which remained dry and untouched for the same periods than the treated biofilms. The experiments included three replicates and six parallel experiments.

### 2.5 Biofilm viability assay of CAP-treated biofilms

*C. albicans* biofilms were treated with CAP as described in Section 2.4. Then 300 μL of a 0.9% NaCl solution was added and shaken at room temperature in orbital motion with the plate shaker (LLG) at 1,500 rpm for 3 min to detach the cells from the surface (Lindsay and Von Holy, 1997). No additional mechanical force (e.g., beads) was used during the detachment procedure. The samples were then serially diluted and inoculated onto Sabouraud maltose agar plates and stored for 24 h at 37°C. The colony count was used to calculate CFU reduction per cm^2^. Each experiment was performed in triplicate with six parallel experiments.

### 2.6 Quantification of respiratory chain dehydrogenase activity in CAP-treated biofilm assay

In the next step, dehydrogenase activity was analyzed for treated and untreated biofilms to assess the effects of CAP on cell metabolism. For this purpose, INT staining [iodonitrotetrazolium or 2-(4-iodophenyl)-3-(4-nitrophenyl)-5-phenyl-2H-tetrazolium] was used to evaluate the activity of respiratory chain dehydrogenase after CAP treatments (1, 3, 5 min) at 8, 12, 16, and 20 V. *C. albicans* biofilms were stained with INT and left in the dark for 24 h at 37°C. Next, INT was reduced by NADH/NADPH and formed a red formazan compound. Subsequently, 150 μL of a 0.9% NaCl solution and 150 μL of DMSO were added to each well of a microtiter plate to release formazan from the biofilm. Subsequently, 200 μL of the suspension was transferred to the 96-well microtiter plate. Formazan was measured with a Sunrise microtiter plate reader (Tecan, Austria) at an optical density of 492 nm. All experiments were performed with three parallel experiments and three replicates.

### 2.7 *C. albicans* cell membrane integrity assessment

To evaluate the effects of CAP on cell membrane integrity, treated and untreated *C. albicans* biofilms were subjected to fluorescent staining with the Live/Dead BacLight Kit (Invitrogen^®^). BacLight fluorescence staining uses two nucleic acid-binding dyes: SYTO 9 (green fluorescent), which penetrates all fungal cell membranes, and propidium iodide (red fluorescent), which only penetrates cells with damaged membranes. Living cells with intact membranes fluoresce green (SYTO 9 only), while dead or damaged ones fluoresce red (propidium iodide displaces SYTO 9) or yellow-orange (mixture of both dyes), allowing differentiation between viable and non-viable cells. The epifluorescent dyes were prepared according to the manufacturer’s instructions. The biofilms were then incubated in the dark for 20 min. After this incubation time, the plates were rinsed with PBS to remove unbound dye. Microscopic analysis was performed using a Motic AE31 Elite fluorescence microscope at 400× magnification and fluorescence dyes (TRIC Ex = 540 nm, Em = 605 nm and MB Ex = 480 nm, Em = 515 nm).

### 2.8 Assessment of intracellular oxidative stress by CAP

The purpose of this method is to quantify total cellular ROS in biofilms using 2′,7′-dichlorodihydrofluorescein diacetate (DCFH-DA) staining. DCFH-DA is a cell-permeable probe that is deacetylated by intracellular esterases to DCFH, which is then oxidized by reactive oxygen species (ROS) to the highly fluorescent compound DCF (dichlorofluorescein). This enables the detection and quantification of intracellular oxidative stress, as the intensity of the green fluorescence is proportional to the amount of ROS present in the cell (Kim and Xue, 2020). CAP-treated and untreated *C. albicans* biofilms were stained with 150 μL DCFH-DA (Sigma Aldrich) at a concentration of 10 μM. This staining procedure was performed in the dark at 37°C for 60 min. Any residual dye was removed by washing with distilled water. To acquire images of the biofilm, fluorescence microscopy was performed using a Motic AE31 Elite fluorescence inverted microscope at excitation and emission wavelengths of MB Ex = 480 nm and Em = 515 nm at 400× magnification.

### 2.9 Morphological assessment of CAP impact on *C. albicans* hyphae

Morphological assessment under the microscope makes it possible to analyze the visual effects of fungal damage. Calcofluor white was used as a fluorescent brightener that binds to chitin and cellulose in the fungal cell walls and makes them fluoresce bright blue-white under UV or blue light excitation. In *C. albicans*, it specifically highlights the cell wall structure and is particularly useful for visualizing fungal morphology, including yeast cells, pseudohyphae and true hyphae, making it valuable for both diagnostic identification and for investigating the integrity of the fungal cell wall. The untreated and CAP-treated *C. albicans* biofilm was stained with 50 μL of Calcofluor white stain. A total of 50 μL of a 10% potassium hydroxide solution was added to each well of the microtiter plate and incubated for 1 min at room temperature. To remove the excess dye, the biofilms were washed with 300 μL PBS. Microscopic analysis was performed using a Motic AE31 Elite fluorescence inverted microscope at 400× magnification and fluorescence dyes (Ex = 355 nm, Em = 433 nm). After staining, the fungal organisms appear light green to blue fluorescent (Merck, 2021).

### 2.10 Biofilm biomass chemical characterization with Fourier transform infrared spectroscopy

The ATR-FTIR spectra (Attenuated Total Reflection integrated Fourier transform infrared spectra) of the *C. albicans* biofilms were recorded before and after CAP treatment for chemical characterization of the biofilm biomass. The aim of the FTIR-ATR analysis was to evaluate the changes in the biofilm matrix and cellular components after CAP treatment by detecting changes in the main functional groups (proteins, lipids, carbohydrates and nucleic acids). This technique allows non-destructive monitoring of the disinfection effect in real time by identifying spectral changes indicative of cell death, membrane damage and biofilm matrix disruption. Using the FTIR method, we compared the permeability of CAP -treated biofilms as a function of wavelength (cm^–1^) using the Agilent Carry 630 FTIR spectrometer. Spectra were collected over the range 3,890–390 cm^–1^ with a resolution of 4 cm^–1^ and an average of 16 spectra per sample. The control samples were left untreated.

### 2.11 Statistical analyses

The software R version 4.2.2. was used for the statistical analysis. Normality was tested using the Shapiro-Wilk test (*p* > 0.05). The Duncan test and the two-way analysis of variance (ANOVA) test were used to determine significant differences. The significance level was *p* < 0.05. Principal component analysis (PCA) was performed in R using the function on vector-normalized, mean-centered, and autoscaled FTIR spectra. Score and loading plots were generated to visualize sample clustering and to identify spectral regions contributing to group discrimination.

## 3 Results

**FIGURE 1:**
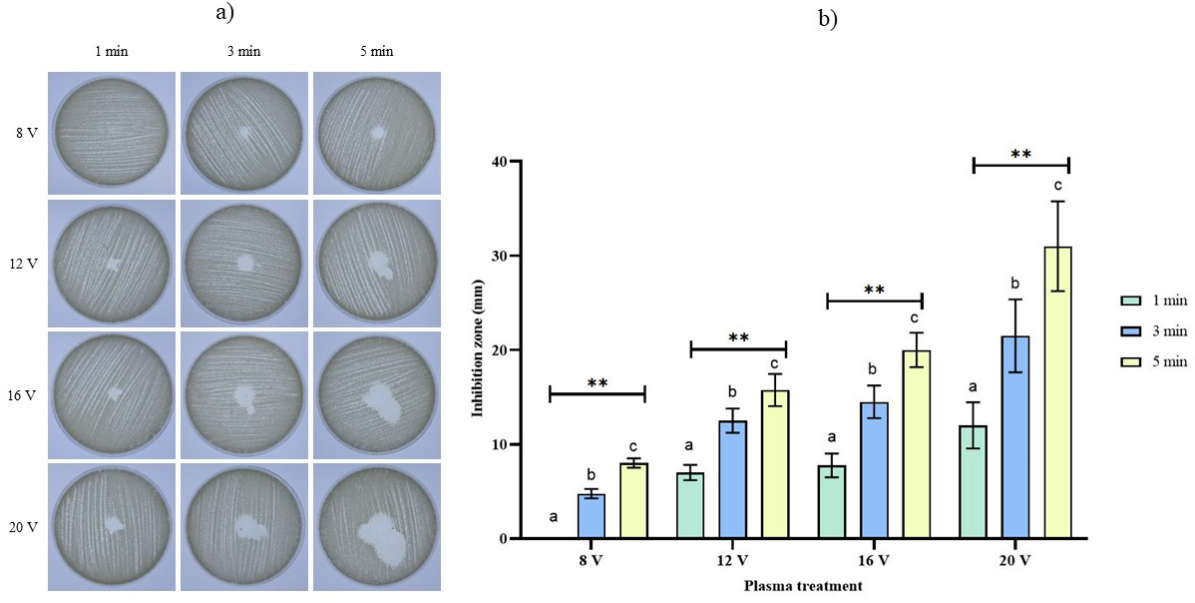
**(A)** Inhibition zones of CAP exposure to *C. albicans* as a function of treatment duration and applied voltages. **(B)** Inhibition diameter (mm) as a function of treatment duration and applied voltages. Data were analyzed using two-way ANOVA followed by Duncan’s *post-hoc* test. Different letters above the columns indicate statistically significant differences for treatment duration (a–c), input voltage (a–c), and their interaction - treatment duration × input voltage (a–c) at *p* < 0.05. **Indicates statistically significant differences at *p* < 0.01.

Firstly, the potential of treating *C. albicans* with CAP was evaluated by analyzing the inhibition zones. The results clearly show that inhibition increases proportionally with increasing treatment duration and increasing electrical voltage (Figure 1A). More specifically, a 1-min treatment at 8 V resulted in no inhibition zone, while a 5-min treatment at 20 V resulted in the maximum measured diameter. At all input voltages, the inhibition diameter increased statistically with treatment duration (*p* < 0.01) (Supplementary Table 1), indicating a CAP parameters-dependent effect of applied voltage and treatment duration (Figure 1B). *Post-hoc* analysis revealed a clear increasing trend of inhibition diameter with increasing time and input voltage treatment (Supplementary Table 2).

**FIGURE 2:**
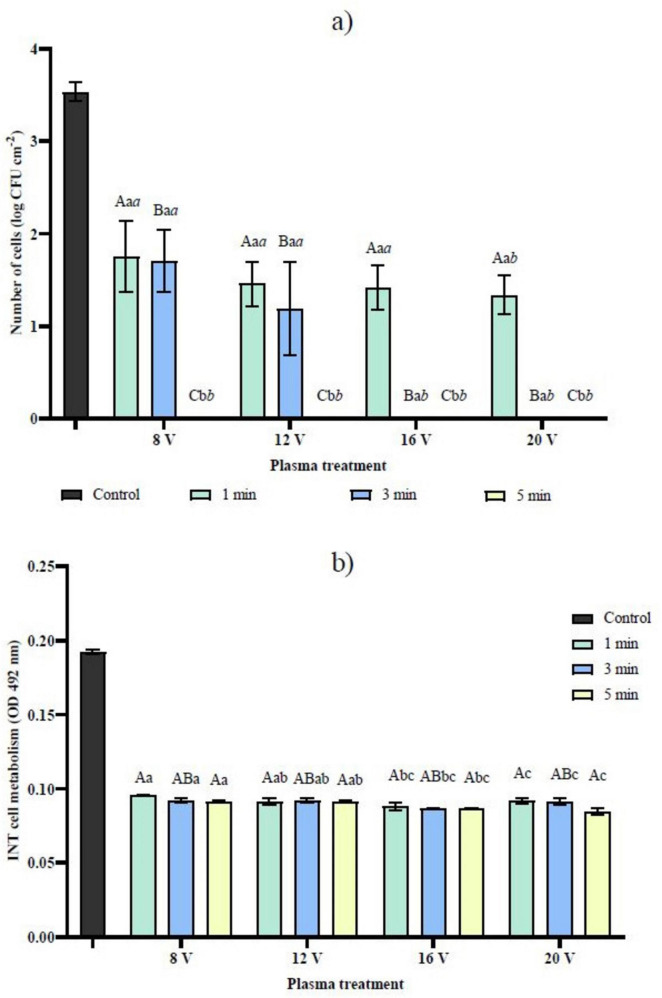
**(A)** Viability and **(B)** respiratory chain dehydrogenase activity of *C. albicans* biofilm before and after treatment with CAP at different treatment duration and input voltages. Data were analyzed using two-way ANOVA followed by Duncan’s *post-hoc* test. Different letters above the columns indicate statistically significant differences for treatment duration (a–c), input voltage (a–c), and their interaction - treatment duration × voltage (*a-c*) at *p* < 0.05.

The viability of the *C. albicans* biofilm was evaluated based on the reduction of CFU after CAP treatment. Efficacy increased with longer treatment duration and higher input voltage, with no fungal growth observed at 8 V and 12 V and 5 min and 3 min at 16 V and 20 V, respectively (Figure 2A). Anova results indicated significant main effects of treatment time (*F* = 109.27, *p* < 0.001) and input voltage (*F* = 64.22, *p* < 0.001) (Supplementary Table 1), as well as a significant treatment time × input voltage interaction (*F* = 16.49, *p* < 0.001) (Supplementary Table 2) on CFU counts. The *C. albicans* respiratory chain inhibition assay using INT assessment showed a significant reduction in dehydrogenase activity even at the lowest input voltage (8 V) and shortest exposure time (1 min). However, longer exposure and higher input voltage (12, 16 V) did not lead to a further significant reduction in OD (Figure 2B). Two-way ANOVA revealed that both treatment time (*F* = 6.37, *p* = 0.020) and input voltage (*F* = 12.02, *p* = 0.002) had a statistically significant effect on INT, while their interaction was not significant (treatment time × input voltage: *F* = 1.18, *p* = 0.291), indicating that the effects of treatment time and input voltage on INT were largely independent. The independence of input voltage or discharge power indicates that the decay of respiratory chain dehydrogenase activity by reactive species from the CAP discharge has already reached a saturation level even at the shortest and mildest treatment conditions.

**FIGURE 3:**
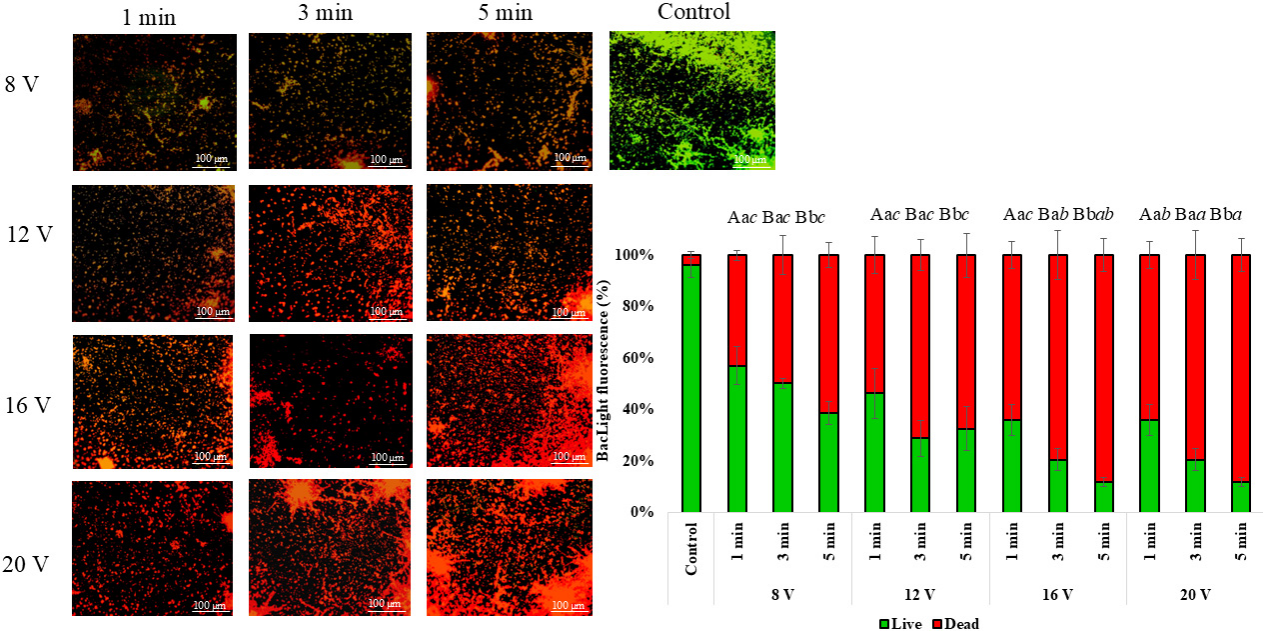
*C. albicans* biofilm integrity of the cell membrane before and after treatment with CAP at different treatment durations and input voltages. Data were analyzed using two-way ANOVA followed by Duncan’s *post-hoc* test. Different letters above the columns indicate statistically significant differences for treatment duration (a–c), input voltage (a–c), and their interaction - treatment duration × input voltage (*a–c*) at *p* < 0.05.

The evaluation of membrane integrity shows a progressive transition from viable to non-viable biofilm populations according to the increasing duration and input voltage of CAP treatment. As shown in Figure 3, the control samples show predominantly green fluorescence, indicating that most cell membranes are intact. At the lowest input voltage setting (8 V), a slight increase in red fluorescence is observed with longer treatment times, indicating initial damage to the cell membranes. As the input voltage increases to 12 and 16 V, the amount of red fluorescence increases significantly, especially with longer exposure times. The most dramatic reaction of the cell membrane occurs at 20 V. Here, extensive red fluorescence dominates the field of view across all treatment times, indicating significant cell death. Quantitative analysis of cell viability shows a clear response relationship between CAP treatment parameters and membrane damage. Under control conditions, cell viability is maintained at about 100%, while CAP treatment leads to progressive damage of the cell membrane, which directly correlates with input voltage intensity and exposure duration. At 8 V, cell viability decreases for about 40% after 1 min and for about 45% after 5 min. Higher voltages lead to much stronger effects on the membrane, with treatments at 20 V leading to a less than 10% cell viability after a longer exposure time. Results show significant effects were observed for treatment time (*F* = 30.65, *p* < 0.001), input voltage (*F* = 263.40, *p* < 0.001), and their interaction (treatment time × voltage: *F* = 6.25, *p* = 0.016), suggesting a strong combined influence of both factors. *Post-hoc* analysis revealed that fluorescence values increased with increasing treatment time and voltage. The highest values were recorded at treatment time = 5 and input voltage = 20, while the lowest occurred at treatment time = 1 and input voltage = 8.

**FIGURE 4:**
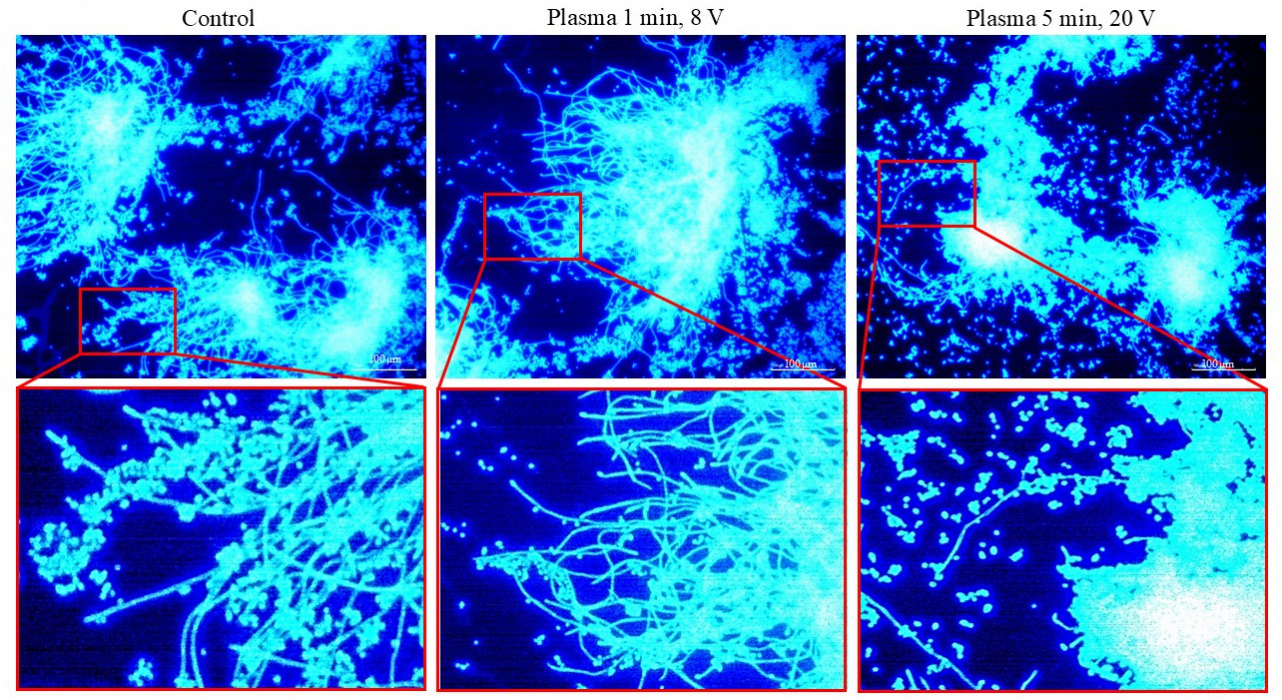
Assessment of the morphology of *C. albicans* with calcofluor white staining before and after treatment with CAP at different treatment durations and input voltages.

The results of the evaluation of the morphology of *C. albicans* before and after treatment with CAP and with the addition of Calcofluor white show different effects on the hyphae. The results show a clear response to the CAP treatment parameters. An increase in treatment duration and applied voltage correlates with increased antimicrobial efficacy and more extensive cellular damage. The progression from mild membrane disruption to complete cell destruction indicates that the efficacy of CAP treatment can be modulated by optimizing the parameters. As can be seen in Figure 4, CAP has a visible effect on the hyphae as they contract and compress together with the cells at longer treatment durations and higher input voltage intensities. Furthermore, the results indicate that CAP treatment induces the *C. albicans* cells to detach from the hyphae.

**FIGURE 5:**
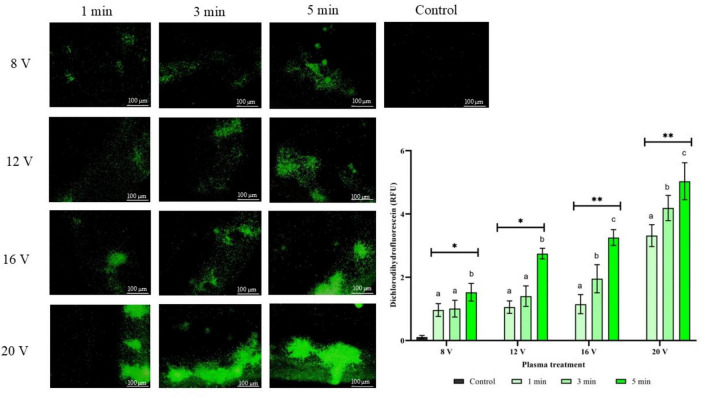
Intracellular oxidative stress of *C. albicans* before and after CAP treatment as a function of different treatment durations and input voltages. Data were analyzed using two-way ANOVA followed by Duncan’s *post-hoc* test. Different letters above the columns indicate statistically significant differences for treatment duration (a–c), input voltage (a–c), and their interaction - treatment duration × input voltage (*a–c*) at *p* < 0.05. *Indicates statistically significant differences at *p* < 0.05. **Indicates statistically significant differences at *p* < 0.01.

The formation of intracellular reactive oxygen species was assessed with the DCFH-DA fluorescence probe after exposure to cold atmospheric CAP at different input voltage settings and treatment durations. Representative fluorescence microscopy images show a progressive increase in green fluorescence intensity corresponding to the increased intracellular ROS concentration with increasing input voltage and exposure duration. Quantitative analysis of fluorescence intensity shows a clear relationship between the CAP treatment parameters and the induction of oxidative stress (Figure 5). At 8 V, a modest increase in ROS generation was observed across all exposure times, with the 5 min treatment showing the most pronounced effect. Higher voltages resulted in much stronger oxidative stress responses, with the 20 V treatment showing the most dramatic increase in fluorescence intensity. The combination of maximum input voltage (20 V) and prolonged exposure duration (5 min) resulted in an approximately 5-fold increase in intracellular ROS levels compared to untreated controls. For DCFH-DA, statistically significant effects were detected for treatment time (*F* = 54.50, *p* < 0.001), input voltage (*F* = 157.56, *p* < 0.001), and treatment time × input voltage (*F* = 4.47, *p* = 0.040), indicating that both factors and their interaction significantly influenced DCF. These results suggest that both input voltage and treatment duration contribute concurrently to cellular oxidative stress and provide clear parameters for the controlled induction of intracellular stress.

**FIGURE 6:**
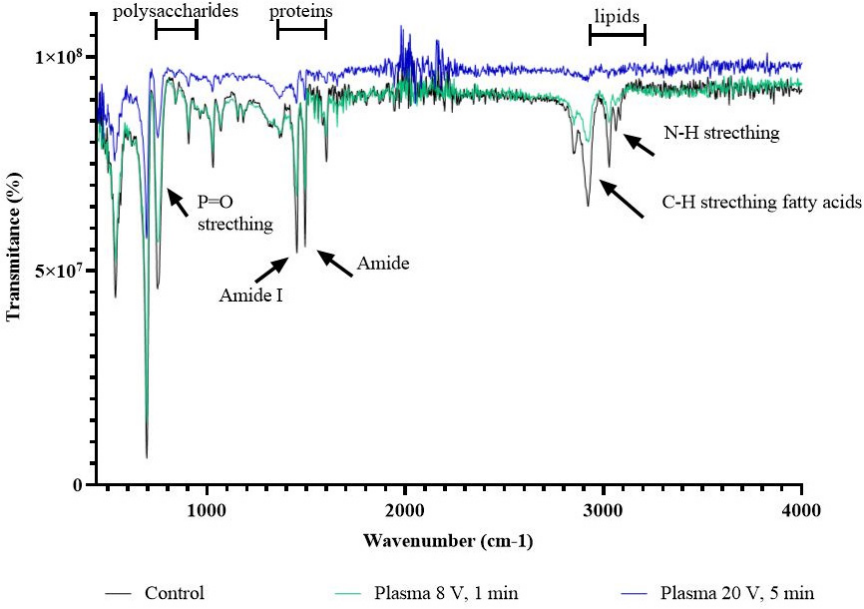
FTIR-ATR analysis of *C. albicans* biofilm before and after treatment with CAP at 8 V, 1 min and 20 V, 5 min.

The results of the FTIR spectrum (Figure 6) show the biochemical changes of biofilm matrix and cellular components after exposure to CAP at different input voltages and durations. The spectrum shows characteristic absorption regions corresponding to the most important biofilm biomolecules. The carbohydrate region (∼900–1,200 cm^–1^) shows complex absorption patterns associated with C-O and C-C stretching vibrations of cellular polysaccharides and glycoproteins. The protein region (ca. 1,400–1,800 cm^–1^) contains several important functional group assignments, including prominent amide I and amide II bands that are diagnostic of protein secondary structure. The lipid region (ca. 2,800–3,000 cm^–1^) exhibits characteristic C–H stretching vibrations of fatty acid chains in cell membranes. A comparative analysis between treatment conditions shows progressive spectral changes that correlate with CAP treatment duration and input voltage. The mild CAP treatment (8 V, 1 min) shows subtle changes compared to the control spectrum, while the intensive treatment (20 V, 5 min) shows more pronounced changes in all biomolecular regions. PCA was applied to the FTIR spectra to objectively evaluate spectral differences among the samples. The first two principal components (PC1 and PC2) explained 70.76% and 29.24% of the total variance, respectively, accounting for 100% of the cumulative spectral variability. The PCA score plot revealed clear discrimination between experimental groups, indicating pronounced biochemical differences. The corresponding loading plots showed that the main contributors to sample separation were spectral regions associated with lipid vibrations (3,000–2,800 cm^–1^), protein-related bands including amide I and II (1,700–1,500 cm^–1^), and carbohydrate-associated vibrations (1,200–900 cm^–1^). These findings suggest that the observed spectral differences primarily reflect alterations in membrane lipid composition, protein structure, and carbohydrate content (Supplementary Table 3 and Supplementary Figure 1).

## 4 Discussion

*Candida albicans* represents a significant public health challenge due to its role as both a common opportunistic pathogen and an increasingly problematic source of healthcare-associated infections (Parambath et al., 2024). The escalating global trend in antifungal resistance has led to a decrease in the efficiency of conventional therapies (Maybin et al., 2023), highlighting the urgent need for new anti-biofilm strategies due to the limited options for treating *Candida*. Although in recent years CAP received intensive scientific interest for bacteria biofilm control (Polito and Kushner, 2024; Wang et al., 2025), less is known about yeast biofilms in context of effects and mechanisms. Our research focused on observing *C. albicans* biofilm in response to varying parameters of CAP application, specifically the influence of treatment duration and input voltage to optimize and tailor biofilm eradication.

In our study, CAP efficiency was initially screened by measuring *C. albicans* inhibition zones post-treatment. This comprehensive assessment of parameters enabled the identification of optimal treatment conditions that can maximize antimicrobial efficacy while minimizing energy consumption and treatment duration. Such optimization is fundamental to the development of CAP assessment of biofilm settings in the next steps of the research. Our initial observation was that inhibition zones became increasingly irregular at higher input voltages. This effect is likely attributable to elevated airflow during treatment. However, this irregularity did not affect subsequent analyses, as those were conducted using microtiter plate-based methods. Longer exposure times consistently increased inhibition zone diameters, aligning with study by Kostov et al. (2015) that tested helium-based CAP jet. Notably, no inhibition zone formation was observed following a 1 min exposure at 8 V, while a 5 min treatment at 20 V resulted in a maximum average inhibition zone diameter of 31 mm. The systematic variation from 8 to 20 V and 1 to 5 min provided sufficient parameter space to establish clear response curves and identify threshold effects with changing the CAP operating parameters.

In the next step, CAP was tested on *C. albicans* biofilms. This approach is critical to confirm the actual antimicrobial efficacy and to determine whether the treatments achieve complete elimination of the pathogen or merely suppress visible growth. Higher input voltages resulted in reduced cell viability, with a complete reduction observed after 5 min of CAP exposure at all tested voltages. Longer treatment durations promote RONS accumulation, disrupting biofilm integrity and causing cell death (Flynn and Gilmore, 2018), higher-voltage CAP generates more pronounced physical and oxidative damage compared with low-voltage CAP (Lunov et al., 2016). Remarkably, no cell viability was observed after 3 min at 16 and 20 V, underscoring the critical role of input voltage in enhancing antimicrobial efficacy. Its importance has also been demonstrated by Zhao et al. (2024), who reported greater reductions of *A. flavus* suspensions with increasing input voltage following dielectric barrier discharge CAP treatment. Similar to our findings, although using a surface microdischarge CAP, Maisch et al. (2012) observed an increasing reductions in *C. albicans* biofilms with longer treatment times. Similarly, Chiodi Borges et al. (2017) also reported a significant decrease in *C. albicans* biofilm viability with increasing exposure duration using a helium-based CAP jet.

The analysis of cell metabolism serves as a sensitive indicator of the effectiveness of the treatment, as it can reveal cell damage prior to visible growth inhibition. This aspect is particularly important for detecting viable but non-culturable (VBNC) bacteria. At low exposures of CAP, bacteria divert most of their energy toward antioxidant defense systems that counteract RONS, thereby entering a dormant VBNC state as a self-protective strategy to adapt to the extreme conditions generated by CAP (Zhang et al., 2023). The ability of VBNC bacteria to regain culturability and proliferate under favorable conditions, such as adequate nutrient availability and optimal temperature, increases the risk of recolonization and contributes to various health problems (Patinglag et al., 2021). In the present study, INT-based metabolic analysis demonstrated a significant reduction in respiratory chain dehydrogenase activity in *C. albicans* biofilms following CAP exposure, even at the lowest tested input voltage (8 V) and shortest treatment time (1 min). These findings indicate that CAP rapidly impairs essential metabolic pathways. Further increases in input voltage parameters (6, 12, and 20 V) did not result in a further reduction in formazan production. Assessment of respiratory chain dehydrogenase activity showed that CAP-generated RONS rapidly saturate cellular metabolic pathways and achieve maximal inhibition even under minimal treatment conditions. This plateau may reflect rapid oxidation or inactivation of key enzymes within the electron transport chain, beyond which additional CAP exposure yields diminishing metabolic suppression (Mahmoud et al., 2025). While CFU counts decreased dramatically at higher voltages and longer treatment times, residual metabolic activity was still detectable by the INT assay. CAP-treated cells that are severely damaged or in a VBNC state may be unable to divide yet retain transient metabolic function, which is captured by INT but not by CFU assays. Similar observations have been reported by Handorf et al. (2018), who tested the use of argon-based radiofrequency plasma jet (kINPen09) and reported an 89% decrease in metabolic activity of *C. albicans* biofilm after 1 min of treatment.

Furthermore, the decrease in viability after CAP treatment was also confirmed by fluorescence staining with BacLight and membrane integrity assessment. CAP is known to trigger a cascade of intracellular reactions, including lipid peroxidation resulting from free radical attack on membrane fatty acids. This oxidative damage compromises membrane structure and permeability, leading to loss of barrier function and cellular homeostasis (Alkawareek et al., 2014). Consistent with this mechanism, increasing CAP treatment time and input voltage produced a progressive shift from predominantly viable to predominantly non-viable cell populations, as indicated by membrane integrity assessment. The majority of cell membranes in the control cells remained intact, as evidenced by their green fluorescence. Longer exposure times at the lowest input voltage setting (8 V) lead to a slight increase in red fluorescence, which could indicate early stage cell membrane damage. The percentage of red fluorescence increases dramatically with increasing input voltage (12 and 16 V), especially with longer exposure times. At 20 V the widespread red fluorescence is also visible across the entire field of view regardless of treatment duration, indicating significant cell membrane damage. These results support findings on viability and metabolism, indicating that 5 min treatment at 16 V input voltage is sufficient for the best antimicrobial effect with the least economical costs. Similar observations were made by Šimončicová et al. (2018) in *A. flavus*, where the negative effects of CAP were observed by the orange/yellow coloration of the cells. Using the viability assay, metabolic activity assay, and BacLight staining a significant toxic impact of CAP on *C. albicans* fungal cells was confirmed.

Furthermore, hyphal growth is a critical virulence factor of the fungus, as the morphological transition from yeast to hyphae is one of the most important virulence factors contributing to the clinical significance of the organism (Gow and Hube, 2012). Our study demonstrates that CAP can damage hyphae, with contraction and compression evident under prolonged treatment and increased CAP stress. Similar results were reported in various studies (Chiodi Borges et al., 2017; Šimončicová et al., 2018; Fink et al., 2023), in which fungal hyphae exhibited roughness, shrinkage and structural damage. Baz et al. (2023) tested *C. albicans* biofilms against argon CAP jet treatment and found that, under the SEM microscope, yeast cells and hyphae were clearly visible in the control biofilms, whereas CAP-treated samples exhibited deflated hyphae, indicating subtle structural deformation. The observed morphological changes are consistent with the multi-targeted antimicrobial mechanism of CAP, as the treatment generates RONS that simultaneously attack multiple cellular components.

Moreover, oxidative stress represents a principal factor governing the cellular response to CAP exposure. Upon treatment, intracellular reactive oxygen species (iROS) rapidly accumulate within microbial cells (Dantas Ada et al., 2015). Although endogenous antioxidant defense systems act to mitigate oxidative damage, higher CAP exposure overwhelms these protective mechanisms, resulting in extensive molecular damage to proteins, lipids, and nucleic acids, ultimately leading to irreversible cellular injury and cell death (Lunov et al., 2017). In the present study, this mechanism was confirmed by the increased green fluorescence observed following reaction with the DCFH-DA probe, indicating elevated iROS levels. Moreover, prolonged treatment durations and higher input voltages led to a gradual increase in iROS accumulation. Similar iROS accumulation following CAP exposure has been reported by Šimončicová et al. (2018) in *A. flavus* mycelium treated with diffuse coplanar surface barrier discharge and by Ma et al. (2013), in *S. cerevisiae* suspension exposed to micro-hollow cathode discharge CAP microjet.

In addition, FTIR analysis of our research showed the effects of CAP on biofilm components, including lipids, proteins and carbohydrates. After a 5-min treatment with 20 V, significant reduction in chemical bonds was observed, indicating cellular damage, biofilm dispersal and effective decontamination. The spectral ranges corresponding to lipids and fatty acids, biomass content, amide I, carbohydrates and cell wall dehydration showed extensive oxidation and degradation. Specific changes in functional groups include variations in P = O stretching vibrations, which may indicate modifications of phospholipid membranes, and shifts in amide bands, which indicate structural perturbations of the proteins. The presence of N-H stretching vibrations in the higher wavenumber range provides additional evidence of protein and nucleic acid interactions with CAP -generated reactive species. These spectral changes show that CAP induces measurable biochemical alterations in biofilm components that correlate with treatment duration and input voltage. Similar results were reported by Soler-Arango et al. (2019) for *P. aeruginosa* and Šimončicová et al. (2018) for *A. flavus*, where CAP reduced important bacterial and fungal components. These results are consistent with our findings on cell viability, membrane activity and reduction of respiratory dehydrogenase and emphasize the efficacy of CAP as an antifungal agent.

## 5 Conclusion

This study evaluates the antifungal activity of CAP against *Candida albicans* biofilms and demonstrates that treatment efficacy varies according to applied voltage and exposure duration. Both parameters contributed to the observed antifungal effects, indicating a parameter-dependent response. Inhibition zone assessment showed a systematic progression of antimicrobial activity ranging from ineffective conditions at 8 V for 1 min to maximum antimicrobial effect at 20 V for 5 min. Evaluation of the viability of colony-forming units showed that complete eradication of the pathogen was achieved at 8 V or 12 V for 5 min and 3 min at 16 V and 20 V, respectively, establishing clear threshold parameters for clinical applications. The mechanistic assessment provided crucial insights into cellular targets affected by CAP treatment. Respiratory chain dehydrogenase activity decreased following CAP exposure, indicating disruption of metabolic activity even at lower input voltages. Membrane integrity assay also demonstrated increasing membrane damage with higher input voltages and treatment durations. Morphological assessment revealed significant structural changes in hyphal architecture, with CAP treatment leading to contractile deformation and detachment of cells from hyphal networks. These results are of particular importance for clinical application, as hyphal formation is a critical virulence factor in the pathogenesis of *C. albicans* and the development of biofilms. Although the 5-min treatment at 20 V visually caused greater hyphal retraction, multiple analyses, particularly CFU counts and membrane damage assays, showed that 16 V treatments at 3 and 5 min achieved comparable antimicrobial effects against *C. albicans*, offering a balance between efficacy and energy consumption. The correlation between the treatment parameters and oxidative stress shows that cellular destruction occurs through a controlled induction of oxidative stress pathways. Biochemical analyses using FTIR spectroscopy confirmed changes at the molecular level in the biofilm components, including changes in the secondary structures of proteins, phospholipid membranes and nucleic acid interactions. Overall, the findings indicate that CAP exerts antifungal effects through a combination of metabolic disruption, membrane damage, oxidative stress induction, and structural alteration. Direct comparison with other studies is however limited by differences in CAP sources, operational conditions, and fungal strains. These results highlight the need to optimize CAP treatment parameters to achieve effective antifungal outcomes.

## Data Availability

The datasets presented in this study can be found in online repositories. The names of the repository/repositories and accession number(s) can be found at: https://doi.org/10.5281/zenodo.12662962.
