# Supplementary material for: Optimizing and tailoring cold atmospheric plasma parameters for C. albicans biofilms eradication
Source: Front Microbiol. 2026 Apr 10;17:1786008. doi: 10.3389/fmicb.2026.1786008 (PMC13106142; doi:10.3389/fmicb.2026.1786008)

# Supplement

## Table S1: Two-way Anova

| ***Inhibition zones*** | | | |
| --- | --- | --- | --- |
| Factor | Df | F - value | *p - value* |
| Time | 1 | 193.53 | <0.001 |
| Voltage | 1 | 300.68 | <0.001 |
| Time ×Voltage | 1 | 23.55 | <0.001 |
| ***Respiratory chain dehydrogenase activity*** | | | |
| Factor | Df | F - value | *p - value* |
| Time | 1 | 6.37 | 0.020 |
| Voltage | 1 | 12.02 | 0.002 |
| Time ×Voltage | 1 | 1.18 | 0.291 |
| ***Cell viability*** | | | |
| Factor | Df | F - value | *p - value* |
| Time | 1 | 109.27 | <0.001 |
| Voltage | 1 | 64.22 | <0.001 |
| Time ×Voltage | 1 | 16.49 | <0.001 |
| ***Intracellular oxidative stress*** | | | |
| Factor | Df | F - value | *p - value* |
| Time | 1 | 54.50 | <0.001 |
| Voltage | 1 | 157.56 | <0.001 |
| Time ×Voltage | 1 | 4.47 | 0.040 |
| ***Cell membrane integrity*** | | | |
| Factor | Df | F - value | *p - value* |
| Time | 1 | 30.65 | <0.001 |
| Voltage | 1 | 263.40 | <0.001 |
| Time ×Voltage | 1 | 6.25 | 0.016 |

Table S2: Post-hoc Duncan test

| ***Inhibition zones*** | | |
| --- | --- | --- |
| Treatment | Average | Group |
| Time 1 | 6.68 | A |
| Time 3 | 13.31 | B |
| Time 5 | 18.62 | C |
| Voltage 8 | 4.16 | a |
| Voltage 12 | 11.75 | b |
| Voltage 16 | 14.08 | c |
| Voltage 20 | 21.50 | d |
| Voltage × Time 20:5 | 31.00 | *a* |
| Voltage × Time 20:3 | 21.50 | *b* |
| Voltage × Time 16:5 | 20.00 | *b* |
| Voltage × Time 12:5 | 15.75 | *c* |
| Voltage × Time 16:3 | 14.50 | *c* |
| Voltage × Time 12:3 | 12.50 | *c* |
| Voltage × Time 20:1 | 12.00 | *c* |
| Voltage × Time 16:1 | 7.75 | *d* |
| Voltage × Time 8:5 | 7.75 | *d* |
| Voltage × Time 12:1 | 7.00 | *d* |
| Voltage × Time 8:3 | 4.75 | *d* |
| Voltage × Time 8:1 | 0.00 | *e* |
| ***Respiratory chain dehydrogenase activity*** | | |
| Treatment | Average | Group |
| Time 1 | 0.09 | A |
| Time 3 | 0.09 | AB |
| Time 5 | 0.08 | A |
| Voltage 8 | 0.09 | a |
| Voltage 12 | 0.09 | ab |
| Voltage 16 | 0.8 | bc |
| Voltage 20 | 0.08 | c |
| ***Cell viability*** | | |
| Treatment | Average | Group |
| Time 1 | 1.27 | A |
| Time 3 | 0.79 | B |
| Time 5 | 0.00 | C |
| Voltage 8 | 1.15 | a |
| Voltage 12 | 0.96 | a |
| Voltage 16 | 0.42 | b |
| Voltage 20 | 0.13 | a |
| Voltage × Time 8:1 | 1.757 | *a* |
| Voltage × Time 8:3 | 1.707 | *a* |
| Voltage × Time 12:1 | 1.575 | *a* |
| Voltage × Time 16:1 | 1.401 | *a* |
| Voltage × Time 12:3 | 1.387 | *a* |
| Voltage × Time 20:1 | 0.402 | *b* |
| Voltage × Time 16:3 | 0.201 | *b* |
| Voltage × Time 12:5 | 0 | *b* |
| Voltage × Time 16:5 | 0 | *b* |
| Voltage × Time 20:3 | 0 | *b* |
| Voltage × Time 20:5 | 0 | *b* |
| Voltage × Time 8:5 | 0 | *b* |
| ***Intracellular oxidative stress*** | | |
| Treatment | Average | Group |
| Time 1 | 1.62 | A |
| Time 3 | 2.14 | B |
| Time 5 | 3.14 | C |
| Voltage 8 | 1.16 | a |
| Voltage 12 | 1.73 | b |
| Voltage 16 | 2.12 | b |
| Voltage 20 | 4.18 | c |
| Voltage × Time 20:5 | 5.037 | *a* |
| Voltage × Time 20:3 | 4.190 | *b* |
| Voltage × Time 20:1 | 3.319 | *c* |
| Voltage × Time 16:5 | 3.257 | *c* |
| Voltage × Time 12:5 | 2.748 | *cd* |
| Voltage × Time 16:3 | 1.955 | *de* |
| Voltage × Time 8:5 | 1.525 | *ef* |
| Voltage × Time 12:3 | 1.403 | *ef* |
| Voltage × Time 16:1 | 1.149 | *ef* |
| Voltage × Time 12:1 | 1.058 | *ef* |
| Voltage × Time 8:3 | 1.009 | *f* |
| Voltage × Time 8:1 | 0.965 | *f* |
| ***Cell membrane integrity*** | | |
| Treatment | Average | Group |
| Time 1 | 17.12 | A |
| Time 3 | 24.55 | B |
| Time 5 | 27.26 | B |
| Voltage 8 | 10.07 | a |
| Voltage 12 | 12.73 | a |
| Voltage 16 | 27.87 | b |
| Voltage 20 | 41.23 | c |
| Voltage × Time 20:5 | 45.731 | *a* |
| Voltage × Time 20:3 | 42.921 | *a* |
| Voltage × Time 16:5 | 38.184 | *ab* |
| Voltage × Time 20:1 | 35.044 | *b* |
| Voltage × Time 16:3 | 31.033 | *b* |
| Voltage × Time 16:1 | 14.403 | *c* |
| Voltage × Time 12:3 | 14.164 | *c* |
| Voltage × Time 12:5 | 12.893 | *c* |
| Voltage × Time 8:5 | 12.258 | *c* |
| Voltage × Time 12:1 | 11.160 | *c* |
| Voltage × Time 8:3 | 10.094 | *c* |
| Voltage × Time 8:1 | 7.883 | *c* |

Table S3: Principal component analysis

|  | **PC1** | **PC2** | **PC3** |
| --- | --- | --- | --- |
| Standard deviation | 36.7254 | 23.6061 | 3.151E-13 |
| Proportion of Variance | 0.7076 | 0.2924 | 0.000e+00 |
| Cumulative Proportion | 0.7076 | 1.0000 | 1.000e+00 |

Figure S1: PC 1 and PC plot


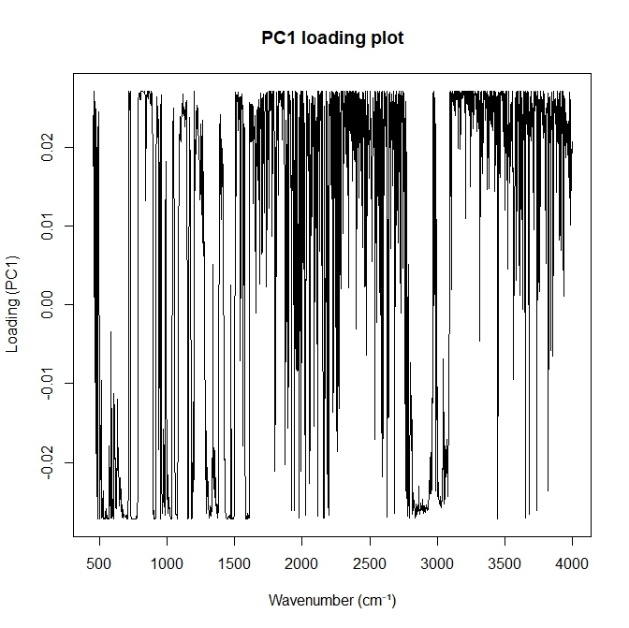

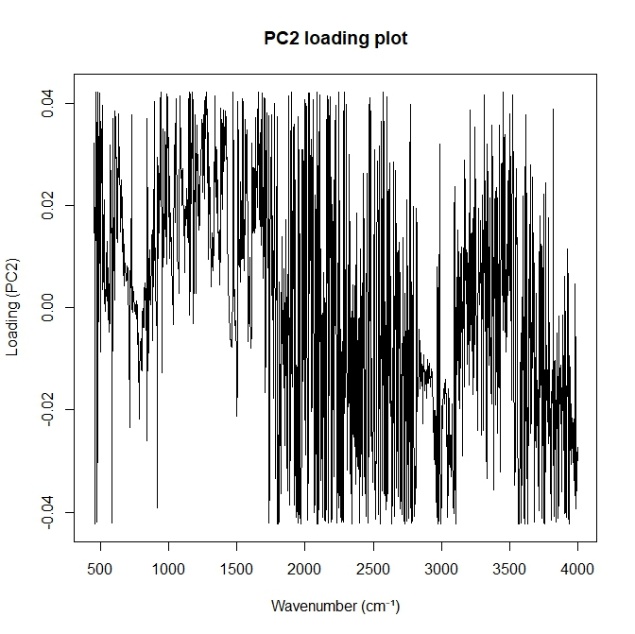

Supplement: Supplementary file 1 [file Table_1.docx]
