# Supplementary material for: Optimizing and tailoring cold atmospheric plasma parameters for C. albicans biofilms eradication
Source: Front Microbiol. 2026 Apr 10;17:1786008. doi: 10.3389/fmicb.2026.1786008 (PMC13106142; doi:10.3389/fmicb.2026.1786008)
Supplement: Supplementary file 3 [file Data_Sheet_1.pdf]

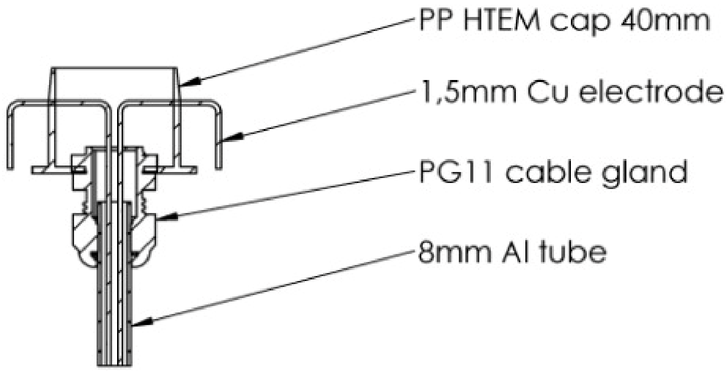

SECTION A-A

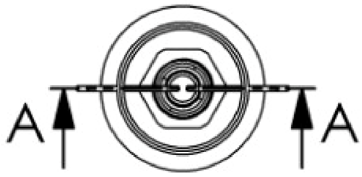

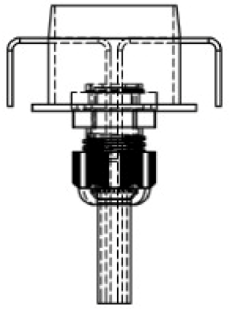

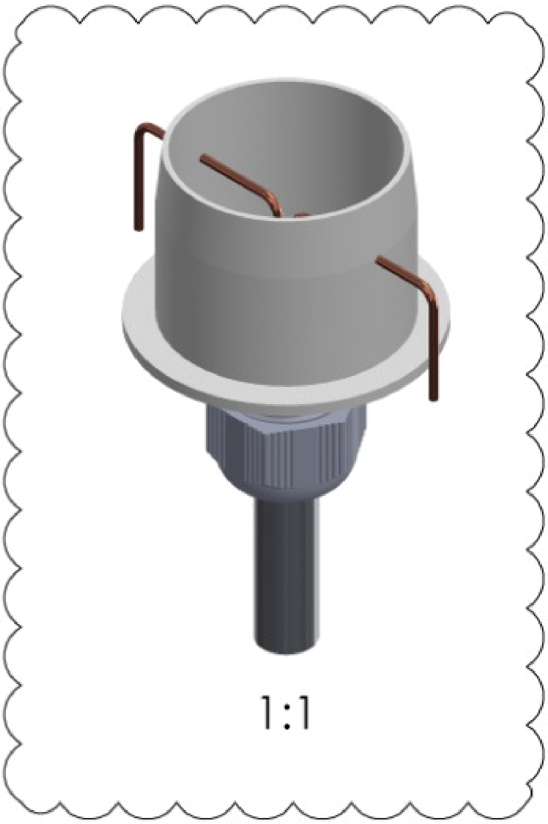

UNLESS OTHERWISE SPECIFIED:
DIMENSIONS ARE IN MILLIMETERS
SURFACE FINISH:
TOLERANCES:
LINEAR:
ANGULAR:

FINISH:

DEBURR AND BREAK SHARP EDGES

DO NOT SCALE DRAWING

REVISION

	NAME	SIGNATURE	DATE
DRAWN			
CHK'D			
APPV'D			
MFG			
Q.A			

TITLE:

MATERIAL:

DWG NO.

Microtiter-Nozzle

A4

WEIGHT:

SCALE:1:2

SHEET 1 OF 1

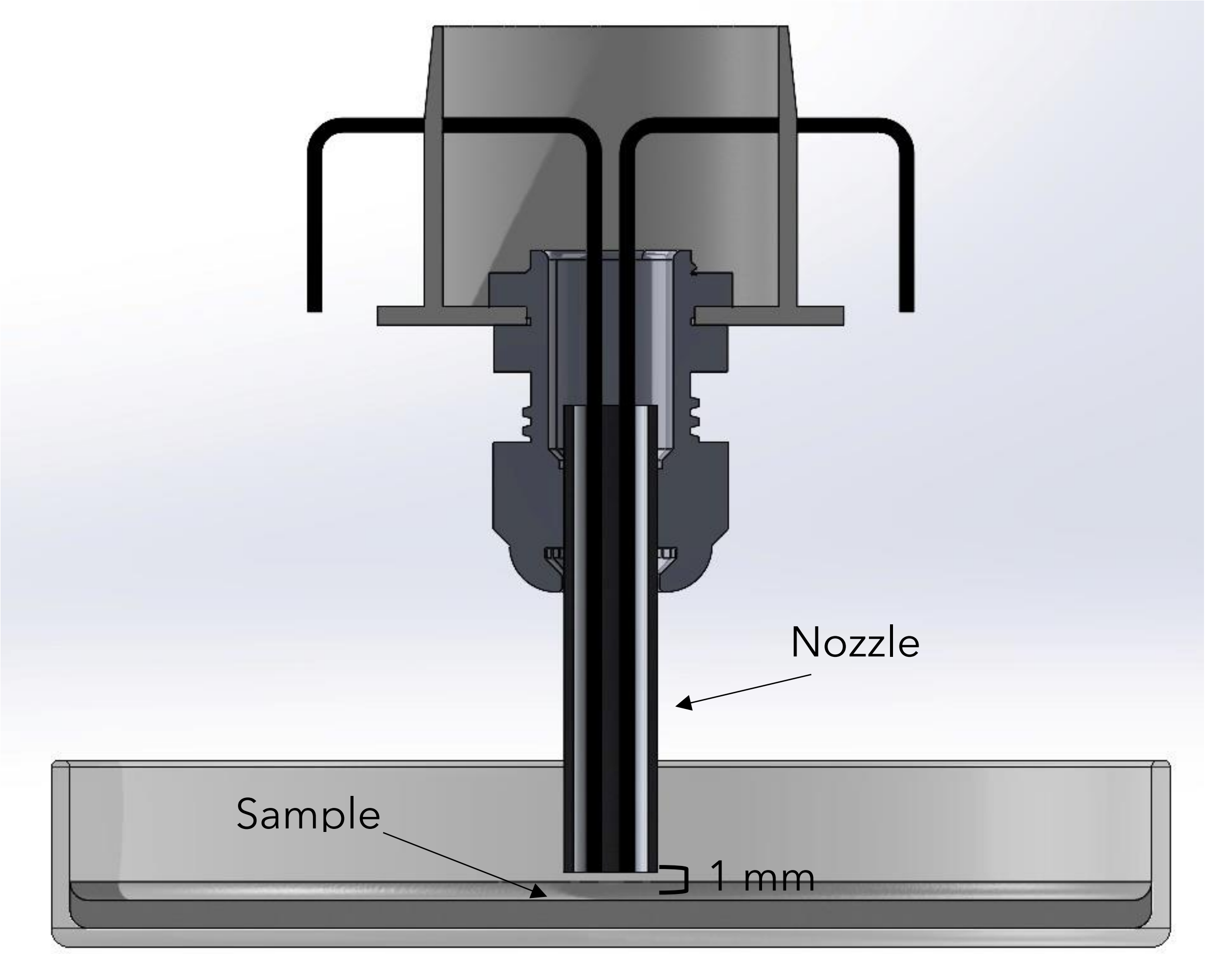

Nozzle
Sample
1 mm
