# Supplementary figures and images for: Optimizing and tailoring cold atmospheric plasma parameters for C. albicans biofilms eradication
Source: Front Microbiol. 2026 Apr 10;17:1786008. doi: 10.3389/fmicb.2026.1786008 (PMC13106142; doi:10.3389/fmicb.2026.1786008)

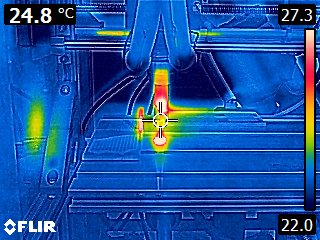

Supplement: Supplementary file 4 [file Image_1.jpeg]
